# Recurrent Tako-Tsubo cardiomyopathy (TTC) in a pre-menopausal woman: late sequelae of a traumatic event?

**DOI:** 10.1186/1471-2261-15-3

**Published:** 2015-01-19

**Authors:** Jochen Hefner, Herbert Csef, Stefan Frantz, Nina Glatter, Bodo Warrings

**Affiliations:** Section of Psychosomatic Medicine and Psychotherapy, Department of Internal Medicine II, Julius-Maximilian-University of Wuerzburg, Oberduerrbacher Str. 6, D- 97080 Wuerzburg, Germany; Unit of Cardiology, Department of Internal Medicine I, Julius-Maximilian-University of Wuerzburg, Wuerzburg, Germany; Comprehensive Heart Failure Center, Julius-Maximilian-University of Wuerzburg, Wuerzburg, Germany; Department of Psychiatry, Psychosomatics and Psychotherapy, Julius-Maximilian-University of Wuerzburg, Wuerzburg, Germany

**Keywords:** Recurrent Tako-Tsubo cardiomyopathy, Chronic distress, Gene-environment interaction, Comprehensive psychosomatic assessment

## Abstract

**Background:**

“Tako-Tsubo cardiomyopathy” (TTC) is a syndrome characterized by left ventricular (LV) wall motion abnormalities, usually without coronary artery disease, mimicking the diagnosis of acute coronary syndrome. It most often affects post-menopausal women and TTC tends to run a benign course with very low rates of recurrence, complications or mortality. The condition is also called “stress-induced cardiomyopathy” because acute physical or emotional stress appears to be frequently related to its onset. The pathogenic role of premorbid or comorbid psychiatric illnesses has been discussed controversially. For the first time, we present a case of fourfold recurrent TTC with severe complications in a pre-menopausal woman. Furthermore, a long history of flaring posttraumatic stress symptoms anteceded the first event.

**Case presentation:**

A 43-year old, pre-menopausal Caucasian woman was hospitalized with symptoms of acute coronary syndrome. Clinical examination revealed hypokinetic wall motion in the apical ventricular region with no signs of coronary artery disease and diagnosis of TTC was established. She experienced recurrence three times within the following ten months, which led to thrombembolism and myocardial scarring among others. The circumstances of chronic distress were striking. 16 years ago she miscarried after having removed a myoma according to her doctor’s suggestion. Since then, she has suffered from symptoms of posttraumatic distress which peaked annually at the day of abortion. Chronic distress became even more pronounced after the premature birth of a daughter some years later. The first event of TTC occurred after a family dispute about parenting.

**Conclusion:**

This is the first case report of fourfold TTC in a pre-menopausal woman. From somatic perspectives, the course of the disease with recurrences and complications underlines the fact that TTC is not entirely benign. Furthermore, it is the first case report of long lasting symptoms of traumatic stress anteceding TTC. Close connections between adrenergic signaling and late onset of clinical stress symptoms are well known in the psychopathology of traumatization. Although larger clinical trials are needed to elucidate possible interactions of premorbid psychiatric illnesses and TTC, cardiologists should be vigilant especially in cases of recurrent TTC.

## Background

Tako-Tsubo cardiomyopathy is a syndrome first described by Sato et al. in 1991 [1] consisting of transient wall motion abnormalities most often involving the apical ventricle. Abnormalities of the electrocardiogram (ECG) and myocardial enzyme release may mimic acute coronary syndrome (ACS) in the angiographic absence of coronary artery disease [2]. The estimated prevalence is 2.5% in patients with ACS and post-menopausal women are most often affected [2, 3]. Usually, TTC subsides rapidly without somatic complications [4]. But a growing number of recent reports demonstrate that TTC is not entirely benign [5, 6]. For example, prolongation of the QT-interval is a well known finding in patients with acute TTC [7]. In a subgroup of patients, the severe prolongation of the QT-interval (QTc > 500 ms) may be a marker for the risk of sudden death [7]. Furthermore, in patients with pre-existing long QT syndrome or concomitant psychiatric diseases and respective medication, TTC may lead to lethal arrhythmias [8]. The in-hospital mortality rate is 1.1% and incidence of recurrence is recently reported to be 2.9 – 10% [4, 9, 10]. Left ventricular thrombus occurs in about 5% of patients, 1.6% suffer from nonfatal cardioembolic outcomes [11]. In a subgroup of patients, cardiac MRI late enhancement may be present and last over time [12]. Late enhancement consistent with myocardial scarring has been reported sporadically and scars were not associated with adverse long term outcomes [5, 13]. The exact pathomechanisms of TTC have not been elucidated [14, 15]. Five different etiological mechanisms of TTC are discussed [16]. There is evidence for (1) multi-vessel epicardial spasms [17], (2) microcirculatory dysfunction [18], (3) obstruction of the left ventricular outflow [19] and (4) endocrine effects like increased vulnerability of postmenopausal women to hormonal and sympathetic stimuli [20]. Most pathophysiological models point to (5) elevated catecholamine levels in TTC patients which are higher compared to patients with myocardial infarction of corresponding Killip classes [21]. In latest reports, a hypothetical association of concurrent sympathetic over activity and vagal withdrawal has been proposed [22]. As acute episodes of physical or mental stress antecede an event in 7 out of 10 cases, stress induced peaks of catecholamine levels acting on differently localized and sensitized adrenergic receptors are thought of setting off an event [2, 23–27]. In several recent reports, a high prevalence of anxiety and depression (21 – 66%) has been found in TTC patients [28–32]. Furthermore, anxiety and depression show effects on catecholamine metabolism. For example, levels of systemic and cardiac catecholamines are elevated and noradrenaline reuptake is reduced in depressed patients [33]. Additionally, noradrenaline responses to emotional stress are correlated with the extent of depressive symptoms [34]. In patients with panic disorders, epinephrine is released from the heart at rest and during spontaneous attacks [35]. Due to a norepinephrine transporter impairment, patients with panic disorder show a reduced uptake of nor-/epinephrine during transit to the heart [36, 37]. In sum, the high prevalence of psychological illnesses in TTC patients led to the assumption that psychosocial stress concomitant with abnormalities in catecholamine signaling may be a risk factor whereas acute physical or emotional stress may ultimately trigger the event [28, 30–32, 38–40]. But the key question of cause and effect of psychiatric disorders in the context of TTC remains. Most of the studies could not determine the timeline of psychiatric disorders, and it may well be that the circumstances after the onset of TTC led to psychiatric comorbidity. In an unprecedented study, a high prevalence (56%) of chronic anxiety disorder anteceding TTC has been reported [32]. The authors hypothesized that premorbid chronic psychiatric conditions may not only be risk factors for TTC but also for recurrences [32].

There is very little documentation of posttraumatic stress disorder (PTSD) in the context of TTC are very rare. At the same time, stressful events antecede the first onset of PTSD and symptoms are often chronic (>90%) and unremitting (>30%) [41]. Hyperarousal is one major clinical sign and the condition is closely linked to neuroendocrine alterations [42, 43]. To our knowledge, there is only one case report of TTC in a postmenopausal patient with a 5 year history of PTSD [44]. The patient recovered fully and there was no report of recurrences or complications [44]. We present a unique case of recurrent TTC with complications in a pre-menopausal woman. Furthermore, it is the first description of chronic symptoms of posttraumatic stress peaking right before the first onset of TTC.

## Case presentation

**Figure 1:**
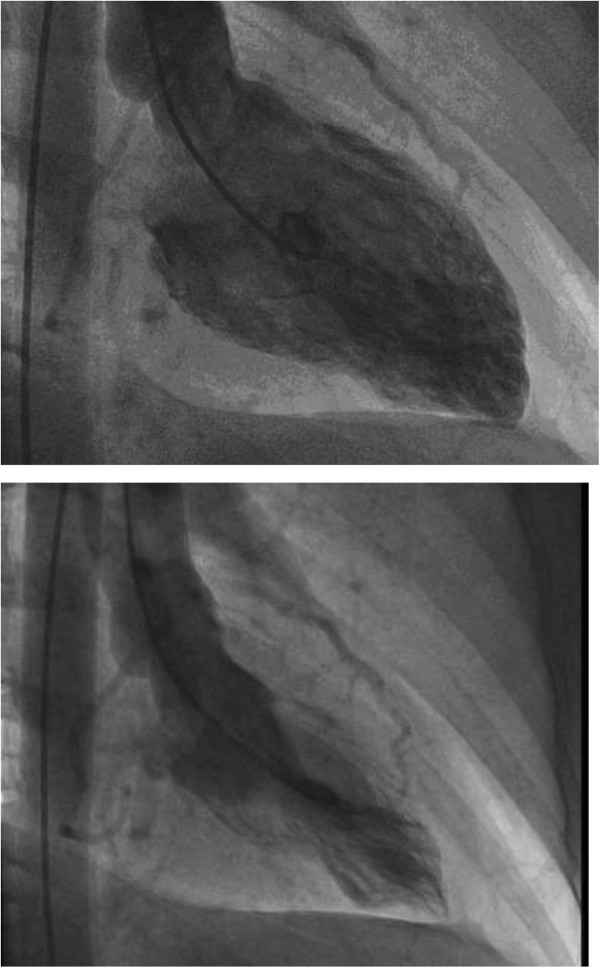
**Left ventriculogram with hypokinetic segments (apical, diaphragmal, and posterobasal) indicative of Tako-Tsubo Cardiomyopathy at first admission to the hospital (upper: diastole; lower: systole).**

A 43 year-old Caucasian woman with a history of hypertension and nicotine abuse was hospitalized with acute onset of chest pain, nausea, and dizziness. Shortly before, she had a vigorous argument with her partner about child education. In the course of the altercation, she was accused to be “a bad mother”. The patient was overwhelmed by the allegations that instantly lead to perceptions of tension, fear and derealisation. At the hospital she appeared anxious with a blood pressure of 168/78 mmHg, a pulse of 78 bpm, shallow breathing and oxygen saturation of 96%. The physical exam and a chest x-ray showed normal findings. Tentative diagnosis was non-STEMI myocardial infarction as ECG was normal and Troponin T levels rose to 0.22 ng/ml. A coronary angiogram revealed no obstructive coronary artery disease or plaque rupture. A left ventriculography demonstrated hypokinetic apical, diaphragmal, and posterobasal segments (Figure 1). The diagnosis of Tako-Tsubo cardiomyopathy (TTC) was established and medication with beta blocker, ACE inhibitor, and aspirin was initiated. An echocardiography five days later showed normal wall movement and an ejection fraction of 72% on echocardiography. The patient was discharged after declining psycho-somatic support.

Six months later, weekly recurrent chest pain increased dramatically while reading a newspaper. Following hospitalization, all results of an examination were very similar to the first event and the diagnosis of recurrent TTC was established.

**Figure 2:**
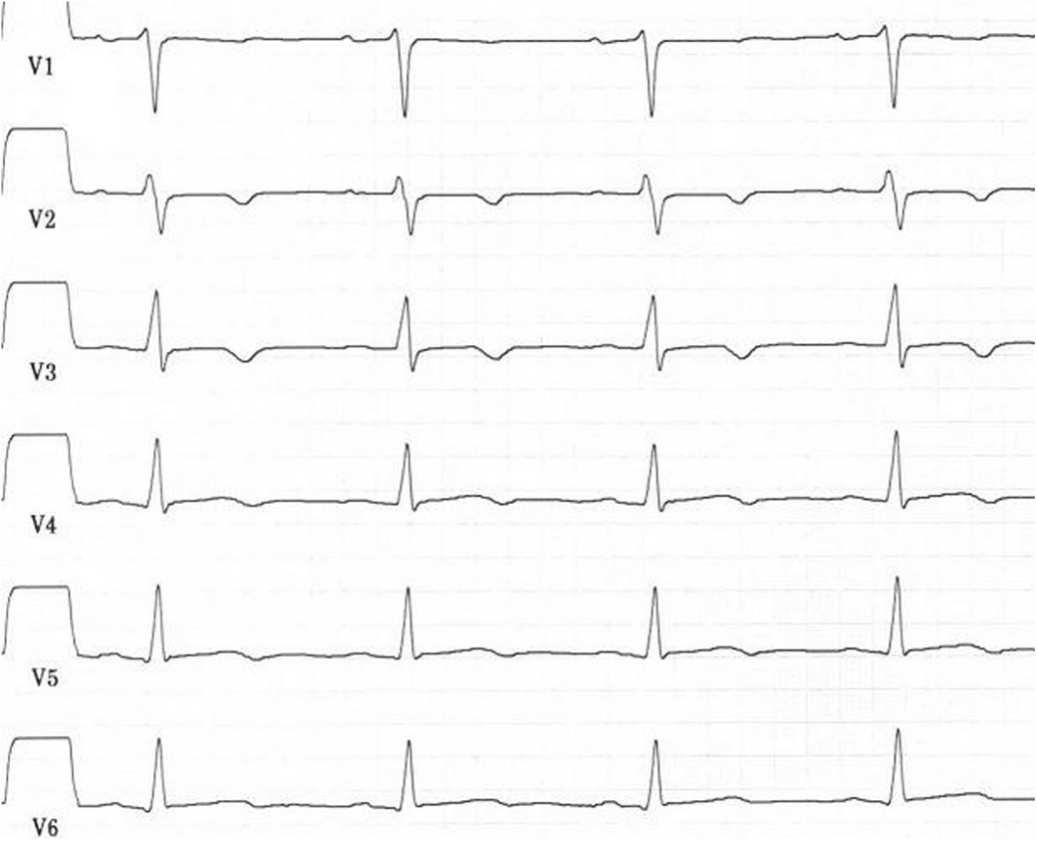
**Electrocardiogram showing T-wave inversions in leads V2-V3 during the second event of Tako-Tsubo cardiomyopathy.**

**Figure 3:**
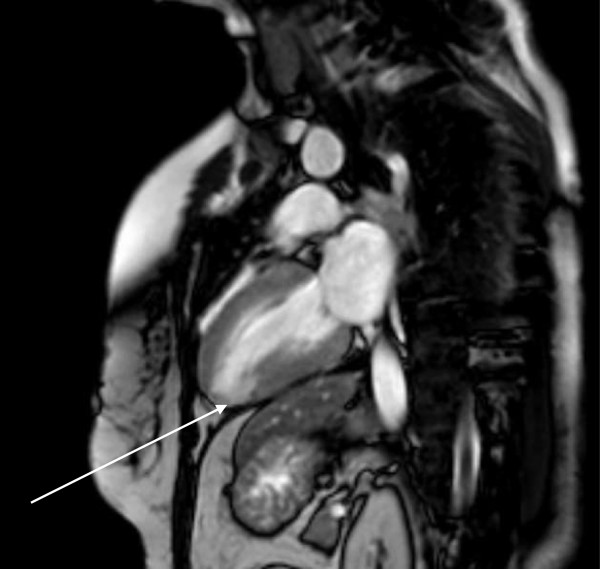
**Cardiac magnetic resonance imaging after fourth event of TTC: transmural scar tissue (inferior apical; white arrow).**

Nine months after the first cardiac event the patient was admitted to the emergency ward again after dyspnoea, chest pain, nausea, and vertigo had increased for several days. This time, the physical exam revealed rales in the right lung field. ECG showed T-wave inversions in leads V2-V3 (Figure 2), and Troponin T levels were at 0.14 ng/ml. Echocardiography demonstrated normal left ventricle function (LVEF > 55%), akinesia of apical segments (septal, lateral, anterior, inferior), and a thrombus of 19 × 11 mm in the left ventricle. Despite administering an anticoagulant, thrombembolism to the right lower leg occurred and had to be surgically removed. After normalized echocardiographic and angiographic results, the patient was discharged a few days later. The last event of TTC emerged another four weeks later. The patient suffered from dyspnoea and pain of the chest and both arms. Physical examination was normal, ECG showed ST depression in leads V2-V4, Troponin T level was 0.06 ng/ml. Cardio-MRI revealed normal left ventricular function (LVEF = 70%) and hypokinetic wall movement of apical and lateral segments. Nonischemic patchy late enhancement was present in the apical, apical inferior septal and lateral regions. A month later late enhancement demonstrated inhomogeneous, inferior-apical transmural scar tissue (Figure 3) which did not change significantly at another follow-up after 9 months. During the fourth event and due to a depressive syndrome, psychosomatic support was once again recommended. This time the patient agreed and for the first time, an antidepressant was administered. Because of latest reports of TTC associated with the use of serotonine and noradrenaline reuptake inhibitors, we selected Mirtazapine in order to avoid increased plasma catecholamine concentrations [45–47]. The psychosomatic history revealed several chronic stress factors. At the age of 27, the patient was pregnant (gestational age 14 weeks) and was advised to undergo surgery of the uterus due to a fast growing myoma. 4 months later fetal death was diagnosed and the pregnancy had to be interrupted shortly before calculated date of birth. This was experienced as a traumatic event by the patient. Since then, she suffered from recurrent solitary symptoms indicative of post traumatic stress disorder (occasional flashbacks, hyperarousal) which did not qualify for a diagnosis of PTSD according to diagnostic manuals. Additionally she described a prolonged grief reaction with rumination about the loss of her child, peaking annually in the month of the fetal death. Three years later, the patient gave birth to a healthy premature child. The patient experienced the incident as a distressing life-event as well and felt chronically concerned about her daughter. Therefore, final diagnoses were recurrent TTC, a chronic posttraumatic stress syndrome with solitary symptoms of PTSD, and a prolonged grief disorder with depressive anniversary reactions.

## Conclusion

Recurrent TTC in pre-menopausal women is very unusual. On the somatic level, a total of four episodes with distinct complications like thromboembolism and scarring of the left ventricle are among the unique peculiarities of this case. Especially the last finding may challenge the diagnosis of TTC. But from our perspective, all symptoms point to unidentified pathomechanisms and to a broader clinical spectrum of TTC [5, 13, 14, 48]. The chronic mental distress of our patient is another remarkable feature of our case. Retrospectively, she suffered from solitary posttraumatic symptoms accompanied by depressive anniversary reactions since the stillbirth 16 years ago. The first event of TTC occurred right after an intense argument about parenting. The psycho-somatic connections underlying TTC are far from clear [14, 15, 39]. In a case study published recently in this journal, Waldenborg et al. even proposed to avoid terms as “stress-induced cardiomyopathy” or “broken heart syndrome” for TTC [49]. They detected symptoms of acute posttraumatic stress in 9 out of 13 (69%) TTC patients (2 patients qualified for having PTSD) and similar numbers of acute distress can be found in patients with acute myocardial infarction [49]. From our point of view, it needs to be taken into account that only one patient had a history of mental illness (manic-depressive disorder), symptoms of borderline or definite PTSD were absent before TTC and recurrences were not reported in a 3 month follow-up [49]. We are aware that the literature on TTC mainly consists of case reports and hypotheses about new pathomechanisms are therefore prone to valid objection. But in line with other researchers we believe that chronic distress may act as a risk factor of TTC and recurrences [32]. Specifically in PTSD, close interactions between trauma and adrenergic signaling do exist [41]. Even on the genetic level, traumatic events may lead to subthreshold endocrine modifications [50, 51]. They in turn may facilitate repeating adrenergic surges many years later [50, 51], even with recurrent cardiac consequences in pre-menopausal women. Despite the practical difficulties, prospective and much larger clinical trials are needed in order to test for these hypotheses. But we believe that already clinicians should be aware of possible connections to long preceding distress or traumatic events especially in cases of recurrent TTC.

## Consent

Written informed consent was obtained from the patient for publication of this case report and any accompanying images. A copy of the written consent is available for review by the Editor of this journal.
